# CD200R1 Supports HSV-1 Viral Replication and Licenses Pro-Inflammatory Signaling Functions of TLR2

**DOI:** 10.1371/journal.pone.0047740

**Published:** 2012-10-17

**Authors:** Roy J. Soberman, Christopher R. MacKay, Christine A. Vaine, Glennice Bowen Ryan, Anna M. Cerny, Mikayla R. Thompson, Boris Nikolic, Valeria Primo, Peter Christmas, Paul Sheiffele, Lisa Aronov, David M. Knipe, Evelyn A. Kurt-Jones

**Affiliations:** 1 Renal Unit, Department of Medicine, Massachusetts General Hospital, Harvard Medical School, Charlestown, Massachusetts, United States of America; 2 Department of Medicine, University of Massachusetts Medical School, Worcester, Massachusetts, United States of America; 3 InGenious Targeting Laboratory Incorporated, Stony Brook, New York, United States of America; 4 Department of Microbiology and Immunobiology, Harvard Medical School, Boston, Massachusetts, United States of America; Emory University School of Medicine, United States of America

## Abstract

The CD200R1:CD200 axis is traditionally considered to limit tissue inflammation by down-regulating pro-inflammatory signaling in myeloid cells bearing the receptor. We generated CD200R1^−/−^ mice and employed them to explore both the role of CD200R1 in regulating macrophage signaling via TLR2 as well as the host response to an *in vivo*, TLR2-dependent model, herpes simplex virus 1 (HSV-1) infection. CD200R1^−/−^ peritoneal macrophages demonstrated a 70–75% decrease in the generation of IL-6 and CCL5 (Rantes) in response to the TLR2 agonist Pam_2_CSK_4_ and to HSV-1. CD200R1^−/−^ macrophages could neither up-regulate the expression of TLR2, nor assemble a functional inflammasome in response to HSV-1. CD200R1^−/−^ mice were protected from HSV-1 infection and exhibited dysfunctional TLR2 signaling. Finally, both CD200R1^−/−^ mice and CD200R1^−/−^ fibroblasts and macrophages showed a markedly reduced ability to support HSV-1 replication. In summary, our data demonstrate an unanticipated and novel requirement for CD200R1 in “licensing” pro-inflammatory functions of TLR2 and in limiting viral replication that are supported by *ex vivo* and *in vivo* evidence.

## Introduction

The host response to viral pathogens is initiated by the interaction of viruses, viral genomes, viral proteins, and viral replication intermediates with host pathogen recognition receptors (PRRs) to trigger the release of cytokines, chemokines, and interferons (IFN) [1], [2], [3], [4], [5], [6]. Ultimately, cells and organisms must integrate input from different signaling pathways in the correct temporal and spatial manner to achieve an appropriate functional state and biological response. In the case of herpes simplex virus 1 (HSV-1) the interaction of the virus with Toll-like receptor (TLR) 2 is critical.

The interaction of TLR2 with HSV-1 triggers NF-κB dependent cytokine production [6], [7], [8], [9] leading to the recruitment and maturation of effector cells for pathogen clearance. Excessive signaling through TLR2 can cause a “cytokine storm”, leading to excessive inflammation and tissue damage resulting in a potentially lethal outcome [7], [9], [10], [11]. In response to HSV-1 infection, TLR2^−/−^ mice show both dramatically enhanced survival and attenuated intracranial generation of CCL5 (Rantes), CCL2 (MCP-1) and IL-6; levels of these cytokines are directly correlated with the severity of HSV-1 infection and the development of immunopathology [9], [12], [13], [14], [15]. Therefore, survival from HSV-1 encephalitis is determined not only by lytic viral infection but also by the balance between pro-inflammatory and down-regulatory anti-inflammatory mechanisms (as well as anti-viral signaling pathways) that limit the pro-inflammatory TLR2 response to infection. However, down-regulatory pathways that interface with TLR2 signaling are poorly described. The HSV-1 model of encephalitis is an ideal model to probe the role of how down-regulatory pathways impact TLR2 biology.

Mouse CD200R1 is a 326 amino acid glycoprotein expressed on the surface of myeloid and glial cells [16]. CD200R1 interacts with CD200, which is expressed on the surface of neurons, epithelial cells, endothelial cells, and lymphocytes [17], . CD200R1 has a 67 amino acid intracellular domain with a unique inhibitory tyrosine motif. Stimulation of CD200R1 by CD200 leads to the phosphorylation of these tyrosines, which recruit the adapter proteins Docking Proteins (Doks) 2 and 1 and SH2 domain-containing phosphatase-1 (SHP-1) to initiate inhibitory signaling and suppress cytokine and chemokine generation [19], [20], [21]. In contrast to CD200R1, the small cytoplasmic domain of CD200 lacks a signaling motif [16], [17]. The interaction of CD200 with CD200R1 down-regulates innate and adaptive immune responses in models of experimental autoimmune encephalitis (EAE), arthritis, transplantation, and acute pulmonary inflammation [16], [17], [18], [22]. However, the specific pro-inflammatory signal transduction pathways in macrophages, dendritic cells, and mast cells that are down-regulated by CD200R1 [19], [20], [21], are poorly described. Specifically, how CD200R1 impacts TLR2 signaling, particularly in macrophages, is not known.

Studies of the role of CD200R1:CD200 interactions in determining the host response to pathogens are complex. To date, they have employed CD200^−/−^ mice, limiting their scope to probing the entire CD200:CD200R1 axis and not capable of detecting other roles for CD200R1 in myeloid cells, i.e., intrinsic functions of CD200R1 independent of CD200 ligand interaction. In the case of influenza and meningococcus, CD200^−/−^ mice are prone to the effects of inflammation/sepsis [18], [23]. This is also the case for *Toxoplasma* encephalitis [24]. In regards to Leishmania, redox defense mechanisms used by the host are suppressed by the CD200R1:CD200 axis in *L. amazonensis*, which induces CD200 expression in host cells, so that CD200*^−/−^* mice are protected from the pathogen. However *L. major* grows more slowly in macrophages and does not induce CD200 expression in host tissue [25]. More recently studies of mouse hepatitis corona virus [26] have shown that the release of inhibition by disrupting the CD200:CD200R1 axis leads to an augmentation of pro-inflammatory signaling and increased recruitment of leukocytes leading to the rapid clearance of virus.

Recently, the concept that inhibitory receptors can be multifunctional and may be required for *pro*-inflammatory signaling has emerged. For example, CD150 contains a TxYxxV/I motif in its cytoplasmic tail that can bind SH2-containing molecules, ranging from tyrosine and inositol phosphatases and Src family kinases to adaptor molecules. The function of CD150 can vary depending on the molecule with which it interacts [27]. CD200R1 has neither a conventional ITIM nor an ITSM domain, and the range of its intracellular functions is not known.

To understand how CD200R1 signaling impacts TLR2 function, we generated CD200R1^−/−^ mice. *In vitro*, we compared the ability of CD200R1^+/+^ and CD200R1^−/−^ macrophages to generate cytokines/chemokines in response to the TLR2 agonist Pam_2_CSK_4_ lipopeptide and to HSV-1. CD200R1^−/−^ macrophages showed a marked decrease in the generation of both IL-6 and CCL5 (Rantes) in response to stimulation by both Pam_2_CSK_4_ and HSV-1, whereas no difference was observed in the generation of any cytokine/chemokine in response to the TLR4 ligand bacterial lipopolysaccharide (LPS). CD200R1^−/−^ macrophages also lacked the ability to up-regulate TLR2 expression in response to HSV-1 infection. CD200R1^−/−^ embryonic fibroblasts and macrophages exhibited a defect in the replication of HSV-1.

*In vivo*, we found that CD200R1^−/−^ mice showed both markedly decreased morbidity and increased survival. CD200R1^−/−^ and CD200R1^+/+^ mice did not show a statistically significant difference in the brain inflammatory response. However, CD200R1^−/−^ mice showed a marked decrease in intracranial HSV-1 titers and expression of HSV-1 envelope glycoproteins. CD200R1^−/−^ showed a decrease in brain levels of IFN-β consistent with the lower viral titers. These results are distinct from those previously observed with TLR2^−/−^ mice, and support a defect in the responses to HSV-1 that include an inability to support viral replication in the absence of CD200R1 in addition to a defect in TLR2 responses.

## Materials and Methods

### Ethics Statement

This study was approved and performed in strict accordance with the guidelines set forth by both the University of Massachusetts Medical School Department of Animal Medicine Institutional Animal Care and Use Committee (IACUC) (assurance number A3306-01) and by the Massachusetts General Hospital (MGH) Center for Comparative Medicine, Subcommittee on Research Animal Care (SRAC), which serves as the Institutional Animal Care and Use Committee (IACUC) at MGH (assurance number A3596-01). Mice were bred and maintained under specific-pathogen-free conditions at the animal facilities at both the University of Massachusetts Medical School and the Massachusetts General Hospital Charlestown Facility, and all efforts were made to minimize suffering.

### Antibodies

Anti-CD200R1 and anti-CD200 for cell surface staining were purchased from AbD Serotec. Goat anti-CD200R1 intracellular domain-specific antibody was from Santa Cruz. Anti-IL-1β was goat polyclonal anti–mouse IL-1β (AF-401-NA; R&D Systems). The primary antibody used in immunohistochemical studies was rabbit polyclonal anti-HSV2 (B0116; Dako) and the secondary antibody used was biotin-conjugated goat anti-rabbit (H+L) IgG (656140; Invitrogen). The secondary anti-rabbit antibody used in blotting studies was rabbit anti-goat (H+L) IgG HRP conjugate (172–1034; Bio-Rad).

### PCR Primers and Screening for CD200R1 Gene Targeting

Mice used were backcrossed to generation N9 or N10. To identify the knockout allele in genomic DNA, tail vein DNA was purified using the DNeasy kit (Qiagen). PCR was then performed using the forward primer Neo1, located in the 5′-promoter region of the *neo* gene cassette (5′-TGCGAGGCCAGGCCACTTGTGTAGC-3′) combined with the reverse primer CD200R1-rev, located outside the short arm of the knockout construct (5′-GGGATGCAGAACATAGGAGGCAG-3′) corresponding to a sequence within the first third of intron 1 of the CD200R1 gene. The PCR product yields a 1.5 kbp product. To identify the wild-type allele primer WT1 (5′-CAGTAGTTTTGGAGAATGTGACAG-3′) was combined with WT-rev (5′-GATAGCCCTTGCTCCTATGACTGAG-3′) to yield a 1.4 kbp product corresponding to a region of intron 1 that is present in WT DNA but is deleted by insertion of the targeting construct. The PCR conditions for both sets of primers were: (95°C, 15 min; 94°C, 30 sec; 62°C, 1 min; 72°C 2 min; 35 cycles using Thermo Start ReddyMix PCR master mix (Thermo Scientific).

#### Exon-Specific screening

The following forward and reverse primers were combined to probe the expression of CD200R1 exons in peritoneal macrophages: exon 1F (5′-ATGTTTTGCTTTTGGAGAACT-3′) and exon 7R (5′-CTAGATTCCAATGGCCGACAA-3′) to amplify the full length cDNA; exon 1F (above) and exon 5R (5′-CACCTCTACTCAGTTCTATGG-3′) to amplify exons 1–5; exon 5F (5′-TGAAGTAACCTACTTTCCAGA-3′) and exon 7R (above) to amplify exons 5–7. RNA was prepared using RNeasy (Qiagen) and RT-PCR was performed using 1 unit Taq polymerase, and Q solution (Qiagen). The PCR conditions for all pairs of primers were: (94°C, 20 sec; 62°C, 20 sec; 72°C 2 min; 35 cycles).

### Preparation and Stimulation of Peritoneal Macrophages

Mice were injected with 4% thioglycollate and peritoneal exudate cells were routinely harvested 3–4 days later [6]. To generate macrophages, peritoneal exudate cells were plated at 10^6^ cells per well in 24-well plates in DMEM containing 10% FCS. Lipopolysaccharide (LPS) was obtained from Sigma and phenol extracted as previously described [28]. Pam_2_CSK_4_ was obtained from EMC Microcollections (Tubingen, Germany).

### Immmunohistochemistry and Viral Particle Detection

Tissues were recovered from mice at necropsy, fixed in Bouin’s solution (Sigma-Aldrich), and embedded in paraffin. For routine histology, 5 µm sections were stained with hematoxylin and eosin. The sections were evaluated by a pathologist without knowledge of the experimental design. For immunohistochemistry, fixed sections from CD200R1^+/+^ and CD200R1^−/−^ mice were deparaffinized, antigen-unmasked using citric acid, probed with antibody to HSV antigens [6], stained with biotin-conjugated secondary antibody, and detected using avidin:biotin-complexed HRP. The localization of HRP-labeled Ab was detected using 3,3′-Diaminobenzidine; hematoxylin was used as a counterstain.

### Histological Scoring of Brains

48 hours post-infection, mice were anesthetized with isoflurane and euthanized by exsanguination. Brains were dissected and stored in Bouin’s Fixative Formula (Fisher Scientific, Pittsburgh, PA) for 24 h. Each brain was cut into 4 coronal sections, paraffin embedded, sectioned, mounted and stained with hematoxylin and eosin (H&E) by the histology core at University of Massachusetts Medical School, Worcester, MA. To quantify the severity and the extent of pathologic inflammation in the brains of HSV-1 infected mice, a histologic inflammation scoring system was used. Each of seven regions of the brain was assigned an inflammation score of 0 (no apparent inflammation), 1 (minimal inflammation), or 2 (above minimal inflammation). Inflammation was judged on the presence and extent of cellular infiltrate and reactive gliosis. The regions of the brain that were scored were: frontal cerebral cortex, posterior cerebral cortex, hippocampus, diencephalon/mesencephalon (which includes the thalamus, hypothalamus and brainstem), cerebellum, the caudal paraventricular structures (defined as those areas adjacent to the lateral ventricles), and the rostral paraventricular structures (defined as those areas adjacent to the 3^rd^ ventricle, the cerebral aqueduct, and the 4^th^ ventricle). Each brain was scored blindly by two observers. The scores for each of the seven regions were summed to arrive at a total brain inflammation score for each mouse with a maximum possible score of 14.

### Generation and Analysis of HSV-1-specific CD8+ T Cells

To analyze the generation of viral-specific CD8+ T cell generation, mice were infected intraperitoneally with 10^4^ PFU of HSV-1. On days 5–9, 50 µl of peripheral blood was withdrawn from tail veins and cells were stained for CD8 antigen using an anti-CD8 APC-conjugated monoclonal antibody, and probed for binding of the SSIEFARL-H2-K^d^-PE gB peptide-(aa 498–505) (gB_498–505_) MHC pentamer [29] to identify HSV-1 specific CD8+ positive T cells [30]. The percentage of CD8 positive cells that were also positive for gB pentamer binding was determined by flow cytometric analysis. (All antibodies were from eBioscience and BD Biosciences, San Diego, CA, while HSV-1 gB^+^_498–505_ pentamer was from ProImmune Inc., Bradenton, FL). Cells were incubated with CD3/CD11b/CD8/gB^+^_498–505_ pentamer and monoclonal antibodies following the manufacturer’s protocol. Stained cells were washed twice with FACS buffer and fixed with BD Cytofix/Cytoperm solution for 20 min at 4°C. Following fixation, the cells were washed twice in BD Perm/Wash buffer, resuspended in 4% paraformaldehyde, and analyzed using a multicolor five-laser LSR II instrument (BD Biosciences, San Diego, CA).

### Preparation of Viruses

HSV-1 strains including viruses expressing ICP8-GFP [31] were generated in the laboratory of Dr. David Knipe. Viruses were added to MEFs and peritoneal macrophages at an M.O.I. of 10∶1.

### Statistical Analysis

An unpaired, two-tailed Student’s t-test or a Kruskal Wallis test was used to determine statistical significance where indicated. Statistics were performed using GraphPad (Prism v5.0d) software. Values of *P*<0.05 were considered significant.

## Results

### Gene Targeting of Mouse CD200R1

We developed a gene targeting strategy to delete the expression of the entire CD200R1 protein including the cytoplasmic tail and transmembrane region (Figure S1) to prevent the formation of a truncation mutant that could function as a dominant negative, potentially disrupting the interaction of other signaling molecules. This strategy was therefore distinct from that of a previously generated CD200R1^−/−^ mouse in which only exons 2–4 (the extracellular domain) were targeted [32]. The targeting vector (Figure S1A) was constructed by using a 1.3 kbp DNA fragment as the short arm. The long arm was a 10 kbp genomic fragment, which starts from EcoRV to SpeI. In this strategy, 1.2 kbp upstream of the ATG start codon, exon 1, and 2.9 kbp of intron 1 were replaced by the Neo gene cassette, resulting in a frame shift mutation and the generation of stop codons in each of the 3 reading frames. To confirm successful gene targeting, DNA was prepared from tail veins and analyzed by both PCR and Southern blot. For Southern blotting, tail vein genomic DNA was digested with Nsi1, resolved by 0.7% agarose gels, transferred to nitrocellulose membranes, and then probed with a 550 bp genomic fragment localized within intron 1 to distinguish targeted and CD200R1^+/+^ DNA. As shown in Figure S1B, DNA from CD200R1^+/+^ mice gave a predicted single band of 7.8 kbp. CD200R1^−/−^ DNA showed a single band of the predicted size of 2.7 kbp. Both bands were detected in DNA from heterozygote mice. When analyzed by PCR, tail vein DNA of CD200R1^−/−^ mice showed a characteristic 1.5 kbp band that was absent in CD200R1^+/+^ DNA (Figure S1C), whereas CD200R1^+/+^ tail vein DNA showed only a 1.4 kbp band. DNA from heterozygous mice showed both bands. Finally, when analyzed by flow cytometry, CD200R1^+/+^, but not CD200R1^−/−^ elicited peritoneal macrophages expressed CD200R1 (Figure S1D).

### CD200R1 Licenses Pro-inflammatory Signaling by TLR2 in Macrophages

**Figure 1:**
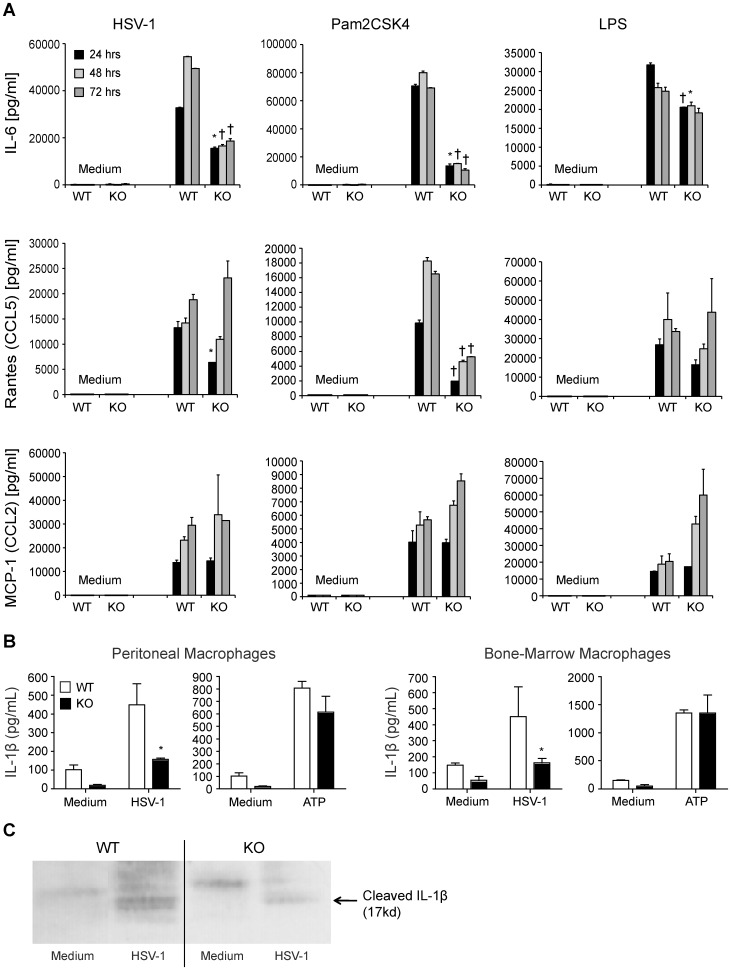
CD200R1 licenses pro-inflammatory signaling in peritoneal macrophages. (A) CD200R1^+/+^ (WT) or CD200R1^−/−^ (KO) elicited peritoneal macrophages were stimulated with either HSV-1 (MOI 10∶1, left column), Pam_2_CSK_4_ (100 ng/ml, center column), LPS (100 ng/ml, right column), or medium for 24, 48, or 72 h. IL-6 (upper row, pg/ml), CCL5/Rantes (middle row, pg/ml), and CCL2/MCP1 (lower row, pg/ml) levels were measured by ELISA. Data shown are mean and SD of representative experiment (total of 3 experiments, n = 4, WT and KO). An unpaired, two tailed Student’s t-test was used to determine statistical significance of independent experiments, p values; * p<0.05, † p<0.01. (B) Supernatant IL-1β levels from WT and KO elicited peritoneal macrophages (left panels) or bone marrow macrophages (right panels) 16 h after addition of HSV-1 (MOI 10∶1) or after a 3 h stimulation with LPS (100 ng/ml) followed by ATP (1 mM) for 1 h. ELISA results are representative of 3 experiments. (C) WT and KO elicited peritoneal macrophages were cultured for 20 h with HSV-1 (MOI 10∶1). Media (left lanes) and cells (right lanes) were analyzed by SDS-PAGE followed by Western blot for cleavage of pro-IL-1β to IL-1β. Data representative of 3 experiments (n = 4, WT; n = 5, KO).

HSV-1 initiates cytokine and chemokine synthesis in the brain via stimulation of TLR2 expressed on macrophages, which are derived from peripheral blood monocytes [6], [7], [8], [9], as well as on glial and microglial cells. We therefore examined the interaction of HSV-1 with CD200R1^+/+^ and CD200R1^−/−^ macrophages. Elicited macrophages from both CD200R1^+/+^ and CD200R1^−/−^ mice were infected with HSV-1 (multiplicity of infection; MOI = 10), or challenged with Pam_2_CSK_4_ (100 ng/ml), or LPS (100 ng/ml), and levels of IL-6, CCL5 (Rantes), or CCL2 (MCP-1) were measured at 24, 48, and 72 h. HSV-1 infected CD200R1^+/+^ macrophages produced 35,000 pg/ml IL-6 24 h after stimulation, which rose to 60,000 pg/ml by 48 h and 72 h (Figure 1A). In contrast, CD200R1^−/−^ macrophages only generated between 20,000 to 30,000 pg/ml IL-6 in response to HSV-1 stimulation. Pam_2_CSK_4_ stimulated IL-6 levels averaged 70,000 to 80,000 pg/ml over the 3 day experiment in CD200R1^+/+^ cells, whereas CD200R1^−/−^ macrophages generated only 10,000 to 20,000 pg/ml. Similar to the IL-6 response, the generation of CCL5 (Rantes) by CD200R1^−/−^ macrophages stimulated with HSV-1 was blunted when compared to CD200R1^+/+^ macrophages. To address whether the decrease in CCL5 (Rantes) was a property of signaling by TLR2, or secondary to low levels of IFN, we determined whether CD200R1^−/−^ peritoneal macrophages exhibit a blunted CCL5 (Rantes) response to stimulation with the TLR4 ligand LPS. In contrast to the attenuated response to TLR2 stimulation, there was only a modest decrease in the levels of IL-6 at 24 h and 48 h and no difference in the levels of CCL5 (Rantes) generated in response to TLR4 stimulation via LPS in CD200R1^+/+^ or CD200R1^−/−^ cells at any time point (Figure 1A). In the case of CCL2 (MCP-1) generation, no difference was found between CD200R1^−/−^ and CD200R1^+/+^ with any stimulant; though LPS induced a marked increase in CCL2 (MCP-1) expression in CD200R1^−/−^ cells.

The formation of mature IL-1β requires two distinct steps [33], [34]. The first signal induces the generation of pro-IL-1β. Expression of pro-IL-1β is regulated at the transcriptional level by NF-κB, which, for HSV-1, is activated downstream of TLR2 signaling. The second signal activates assembly of the inflammasome complex and cleavage of pro-IL-1β into its mature secreted form, IL-1β. This can be triggered by a variety of mechanisms, including extracellular ATP acting on the P2X receptor, reactive oxygen species, or potassium efflux triggered by the antibiotic Nigericin [35]. The interaction of double stranded DNA and viral replication intermediates with intracellular DNA sensors can also induce inflammasome assembly [3], [36], [37], [38], [39], [40]. The assembly and function of the inflammasome is therefore dependent on the quantity of viral replication intermediates within cells. We therefore hypothesized that if CD200R1 was required for efficient viral replication, then CD200R1^−/−^ macrophages should show lower levels of inflammasome formation and impaired conversion of pro-IL-1β to IL-1β.

Stimulation of CD200R1^−/−^ peritoneal or bone marrow-derived macrophages with HSV-1 led to the production of approximately 20% of the mature IL-1β levels seen in CD200R1^+/+^ cells (Figure 1B). In contrast, no difference was found in mature IL-1β levels between CD200R1^+/+^ or CD200R1^−/−^ macrophages stimulated with LPS (100 ng/ml) for 3 h followed by the addition of ATP (1 mM) for 1 h. These results suggested that that there was no intrinsic defect in inflammasome formation or in the capacity of CD200R1^−/−^ macrophages to generate pro-IL-1β mRNA while virus-induced inflammasome formation was significantly attenuated in CD200R1^−/−^ macrophages. The difference in the production of mature IL-1β between CD200R1^+/+^ and CD200R1^−/−^ macrophages following HSV-1 infection were confirmed by Western blot (Figure 1C).

### CD200R1 Expression Regulates TLR2 Expression in Macrophages

**Figure 2:**
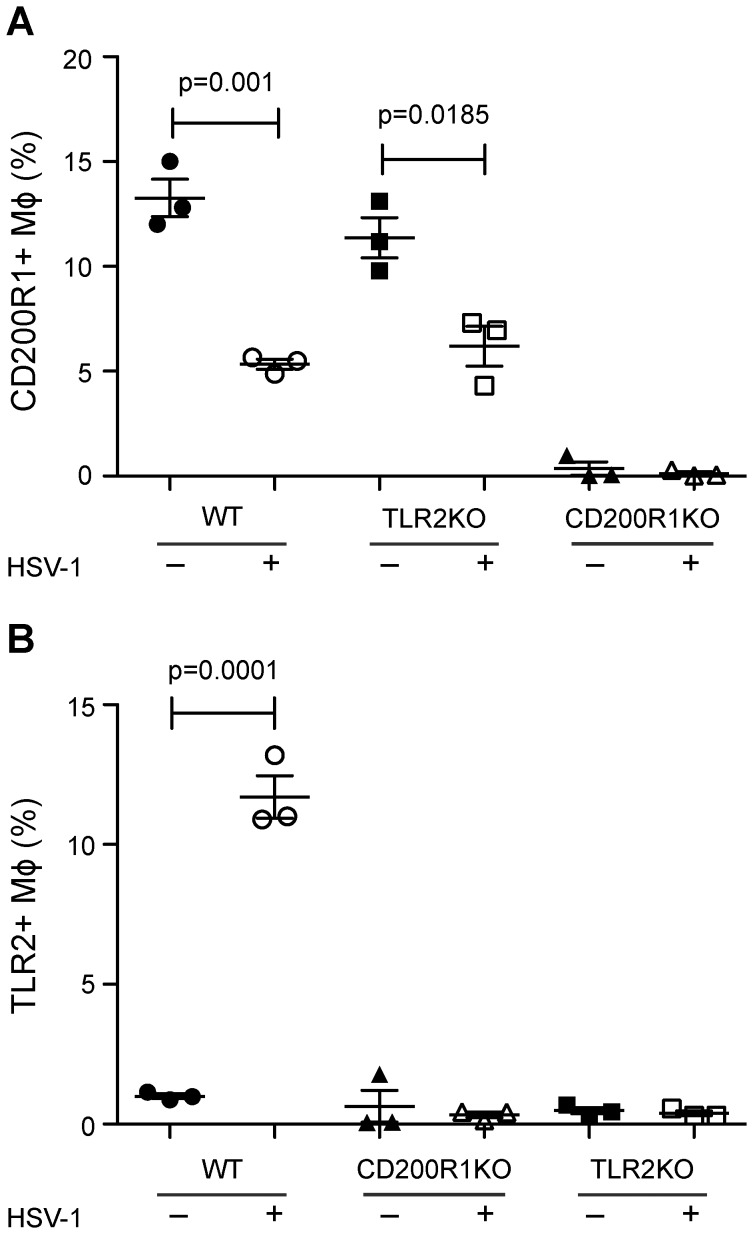
CD200R1 controls expression of TLR2 in macrophages. (A, B) The expression of CD200R1 (A) or TLR2 (B) on the surface of WT (circles), TLR2KO (squares), or CD200R1KO (triangles) elicited peritoneal macrophages (MΦ) before (–, closed symbols) or after HSV-1 infection (+, open symbols) was determined by flow cytometry. The lack of expression of CD200R1 or TLR2 on the surface of CD200R1KO or TLR2KO macrophages, respectively, served as internal controls. Data representative of 3 experiments (preparations from individual mice n = 3 WT and KO are shown). An unpaired, two tailed Student’s t-test was used to determine statistical significance of independent experiments.

Because of the key role of TLR2 in mediating the response of macrophages to HSV-1 [6], [9], we examined the relationship between TLR2 expression on the surface of peritoneal macrophages, CD200R1 expression, and HSV-1 infection. The expression of CD200R1 on the surface of WT and TLR2^−/−^ elicited macrophages was determined by flow cytometry 24 h after infection with HSV-1 (Figure 2A). As an internal control, CD200R1^−/−^ cells were also included. There was no difference in CD200R1 expression levels between TLR2^+/+^ and TLR2^−/−^ macrophages. Interestingly, the surface expression of CD200R1 decreased 24 h after HSV-1 infection in both WT and TLR2^−/−^ macrophages (Figure 2A). This was contrasted with the effect of CD200R1 on TLR2 expression. Following HSV-1 infection, TLR2 expression levels increased 12-fold in WT cells, but TLR2 expression was not increased in CD200R1^−/−^ macrophages (Figure 2B). These results indicated that CD200R1 directly controls the inducible surface expression of TLR2 either directly or secondary to decreased viral replication and that it may play a role in the amplification of HSV-1 infection.

### CD200R1^−/−^ Mice are Protected from Intracranial HSV-1 Infection

**Figure 3:**
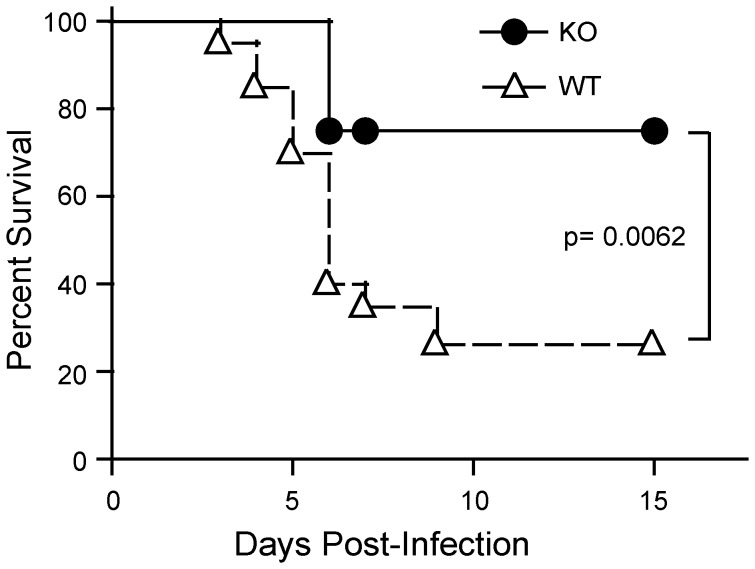
CD200R1^−/−^ mice are protected from HSV-1 encephalitis. CD200R1^+/+^ (WT) and CD200R1^−/−^ (KO) mice were administered 10^5^ PFU HSV-1 by intracranial injection. Survival was monitored for 15 days. Data includes a total of 3 separate experiments (n = 20, WT; n = 16, KO). An unpaired, two tailed Student’s t-test was used to determine statistical significance.

The CD200R1:CD200 axis is known to down-regulate intracranial inflammation, typified by studies of the EAE model [17], [18]. Because morbidity and mortality to HSV-1 infection is secondary to increased brain inflammation (as well as lytic virus replication), we predicted that CD200R1^−/−^ mice would have a marked increase in both brain inflammation and mortality when compared to CD200R1^+/+^ mice. To examine the effect of the CD200R1:CD200 axis in the intracranial inflammation associated with HSV-1 infection, CD200R1^+/+^ and CD200R1^−/−^ mice were infected with 10^5^ PFU of HSV-1 by intracranial injection, and the survival in each group was followed daily for 15 days. CD200R1^+/+^ mice showed a characteristic survival curve (Figure 3), with 40% of the mice surviving by day 6 and 26% by day 9. No further mortality after day 9 was observed. In contrast, CD200R1^−/−^ mice exhibited significantly higher survival rates, with 75% of the mice surviving on day 6, and no further deaths within the course of the experiment.

### Cytokine/chemokine Levels in the Brains of CD200R1^+/+^ and CD200R1^−/−^ Mice is Indistinguishable after Intracranial HSV-1 Infection

**Figure 4:**
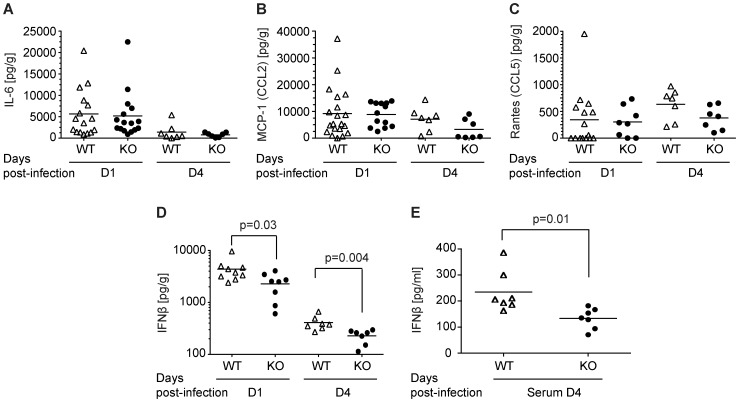
CD200R1^−/−^ and CD200R1^+/+^ brains have distinct cytokine response profiles. (A-D) Cytokine levels were measured in CD200R1^+/+^ (WT, open triangles) and CD200R1^−/−^ (KO, closed circles) brains on days 1 and 4 (D1 and D4) post-cranial injection of 10^5^ PFU HSV-1. (A) IL-6; (B) CCL2/MCP-1; (C) CCL5/Rantes; (D) IFN-β. The mean of each group is indicated by a horizontal bar. The statistically significant groups, as determined by Kruksal Wallis test, are linked by bars and have p-values listed. (E) IFN-β levels were measured in the serum of CD200R1^+/+^ (WT, open triangles) and CD200R1^−/−^ (KO, closed circles). Statistical analysis was performed as above. Data includes a total of 5 experiments each with 3–7 mice per genotype (n = 20 WT and n = 13 KO day 1; n = 7, WT and KO day 4).

To explain these apparently paradoxical results, we examined a series of parameters that define intracranial inflammation associated with HSV-1 infection. CD200R1^+/+^ and CD200R1^−/−^ mice were infected with HSV-1 and their brains were harvested and analyzed to assess inflammation 1 and 4 days post-infection. Brains were homogenized and intracranial levels of three cytokines known to be associated with and/or causative for HSV-1 mediated brain inflammation and that are also linked with the outcome of HSV-1 encephalitis, IL-6, CCL5 (Rantes) and CCL2 (MCP-1) [6], [9], [12], [13], [14], [15] were measured. Levels of IL-6 are known to correlate with the level of viral infection [12], [15], and IL-6^−/−^ mice have more severe HSV-1 encephalitis, with increased morbidity and death than IL-6^+/+^ mice [12]. Levels of IL-6 were statistically indistinguishable between CD200R1^+/+^ and CD200R1^−/−^ brains on day 1, and were essentially at baseline levels by day 4 (Figure 4A). Similarly, CCL5 (Rantes) and CCL2 (MCP-1) levels were not statistically different between CD200R1^+/+^ and CD200R1^−/−^ brains on day 1 or day 4 (Figures 4B and 4C), though the differences in Rantes (CCL5) and MCP-1 (CCL2) on day 4 were trending towards significance with p-values of p = 0.09 and p = 0.07, respectively.

### HSV-1 Induced Type I IFN Production is Significantly Lower in CD200R1^−/−^ Mice

During the initial phase of HSV-1 disease, viral replication is controlled to a large extent by the production of type I IFN and the expression of IFN-response genes [9], [41], [42], [43], [44], [45], [46]. Therefore, we determined levels of IFN-β in the brains of CD200R1^−/−^ and CD200R1^+/+^ mice following intracranial HSV-1 infection. On day 1, CD200R1^−/−^ brains had significantly lower levels of IFN-β than CD200R1^+/+^ mice (Figure 4D). On day 4, the levels IFN-β in CD200R1^−/−^ remained statistically lower than CD200R1^+/+^ mice, though both exhibited a decrease from day 1 levels. In addition, serum IFN-β levels in CD200R^−/−^ mice were 76% lower compared to the levels found in CD200R1^+/+^ mice.

### CD200R1^−/−^ Mice Have Lower Brain Viral Titers than CD200R1^+/+^ Mice

**Figure 5:**
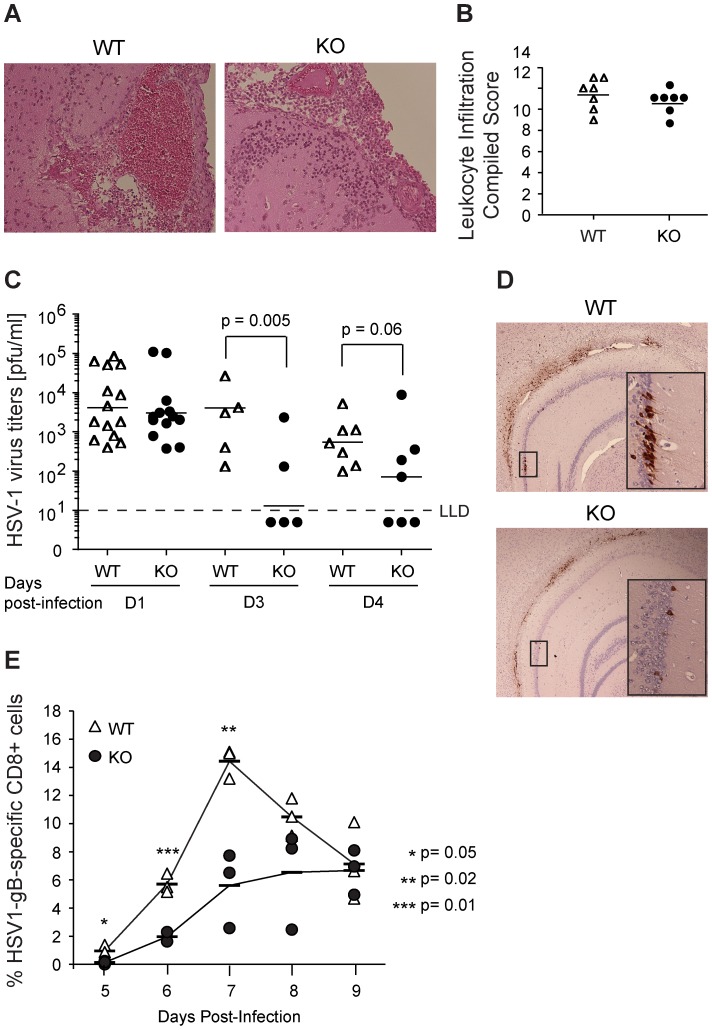
CD200R1^−/−^ mice clear HSV-1 virus more efficiently than CD200R1^+/+^ mice. (A) H&E staining of CD200R1^+/+^ (WT) and CD200R1^−/−^ (KO)^-^ brain sections prepared on day 3 post-intracranial infection (10×). Data is representative of 2 separate experiments each with 3–5 mice per genotype (n = 7 WT and n = 8 KO). (B) Total leukocyte infiltration scores from 7 regions of the brain on day 3 post-intracranial infection. (C) HSV-1 titers in WT (open triangles) and KO (closed circles) brain homogenates on days 1, 3, and 4 post-intracranial infection by plaque assay. Limit of detection (LLD) indicated by dashed line. Data includes a total of 4 experiments (n = 14, WT day 1; n = 13, KO day 1; n = 5, WT and KO day 3; n = 7, WT and KO day 4). (D) HSV-1 antigen expression in WT and KO infected brains (10×). Inset images are 40X zoom of boxed areas. Data includes 2 experiments (n = 6 WT and KO). (E) WT (open triangles) and KO (closed circles) CD8+ T cells were probed for binding of the SSIEFARL-H2-K^d^-PE gB peptide-(aa 498–505) MHC pentamer to identify HSV-1 specific CD8+ T cells on days 5–9 post-intraperitoneal infection. The mean of each group is indicated by a horizontal bar. Statistically significant groups (as determined by an unpaired, two-tailed Student’s t-test analysis) are linked by bars, or indicated by asterisks and have p-values listed. Data are from n = 3, WT and KO.

No difference in gross pathology between CD200R1^+/+^ and CD200R1^−/−^ brains was observed on day 3 post HSV-1 infection (Figure 5A). Leukocyte infiltration was assessed in 7 regions of the brain and each region received a score of 0 (no leukocytes), 1 (rare or occasional leukocyte infiltration) or 2 (marked leukocyte infiltration). Total scores were determined in a blinded manner by 2 observers. Both CD200R1^+/+^ and CD200R1^−/−^ brains showed indistinguishable degrees of leukocyte infiltration in both regional and compiled pathologic scores (Figure 5B).

The lack of difference in brain leukocyte scores, and in CCL2 (MCP-1), CCL5 (Rantes), and IL-6 levels indicated that CD200R1^−/−^ mice had equivalent or modestly reduced brain inflammation, rather than an enhanced, inflammatory response to HSV-1 infection compared to CD200R1^+/+^ mice (see above). When combined with the observation that CD200R1^−/−^ mice had lower levels of IFN-β in brain and serum, the results suggest that there is decreased replication of HSV-1 in CD200R1^−/−^ brains. To test this conclusion, CD200R1^+/+^ and CD200R1^−/−^ mice were infected intracranially with 10^5^ PFU HSV-1, and virus titers in whole brain homogenates were determined by plaque assay using Vero cells on days 1, 3, and 4 post-infection (Figure 5C). On day 1, brain titers were comparable between CD200R1^+/+^ and CD200R1^−/−^ mice. Though viral titers increased in both mice on day 2, CD200R1^−/−^ mice exhibited significantly lower titers than CD200R1^+/+^ mice (not shown). HSV-1 titers were negligible in CD200R1^−/−^ brains by day 3, with 60% of CD200R1^−/−^ mice having brain titers that were below the limit of detection. In contrast, HSV-1 titers from CD200R1^+/+^ brains were unchanged from day 1 to day 3. On day 4, viral titers from CD200R1^+/+^ brains had dropped slightly compared to their titers on day 3 but this was not statistically different. CD200R1^−/−^ mice, however, continued to have a high percentage of animals (42%) that had completely cleared the infection. Overall, the HSV-1 titers in CD200R1^−/−^ mice were lower compared to CD200R1^+/+^ mice on day 4 but the difference in titers did not reach statistical significance. Importantly, approximately 50% of the CD200R1^−/−^ mice had cleared the infection by days 3 and 4, while HSV-1 continued to replicate at high levels in 100% of CD200R1^+/+^ mice.

To assess the extent and distribution of HSV-1 infection in CD200R1^−/−^ and CD200R1^+/+^ brains, we determined the expression of HSV-1 viral antigens by immunohistochemical staining 3 days post-intracranial infection. HSV-1 antigens were detected in a large percentage of neurons in and around the hippocampus of CD200R1^+/+^ mice (Figure 5D). In contrast, CD200R1^−/−^ mice had much less extensive neuron involvement with fewer HSV-1 positive neurons in the hippocampal region.

In response to HSV-1 infection, the host generates CD8^+^ cells that recognize the HSV-gB protein. The HSV-1-specific CD8+ response is dependent on the viral load [29], [30]. A majority of the CD8+ cells are directed to a single dominant gB498–505 peptide and will specifically bind a MHC-gB-peptide-pentamer that can be monitored by flow cytometry [29], [30], [47], [48], [49]. CD200R1^+/+^ and CD200R1^−/−^ mice were infected with 10^4^ PFU HSV-1 by intraperitoneal injection. The development of HSV-gB pentamer-specific CD8^+^ peripheral blood T cells was monitored up to 9 days post-infection (Figure 5E). In CD200R1^+/+^ mice, the percentage of gB-pentamer positive cells peaked 7 days after infection at approximately 14.4%, which decreased by half by day 9 (Figure 5E). In contrast, CD200R1^−/−^ mice showed a blunted and delayed response that gradually rose to approximately 6.5% by day 9. This blunted CD8+ response is consistent with reduced HSV-1 replication in CD200R1^−/−^ mice and provide additional evidence that CD200R1^−/−^ mice either more efficiently eliminate HSV-1 or lack the ability to support its sustained replication.

### CD200R1 Expression Increases HSV-1 Infection *ex vivo* in Mouse Embryonic Fibroblasts and Peritoneal Macrophages

**Figure 6:**
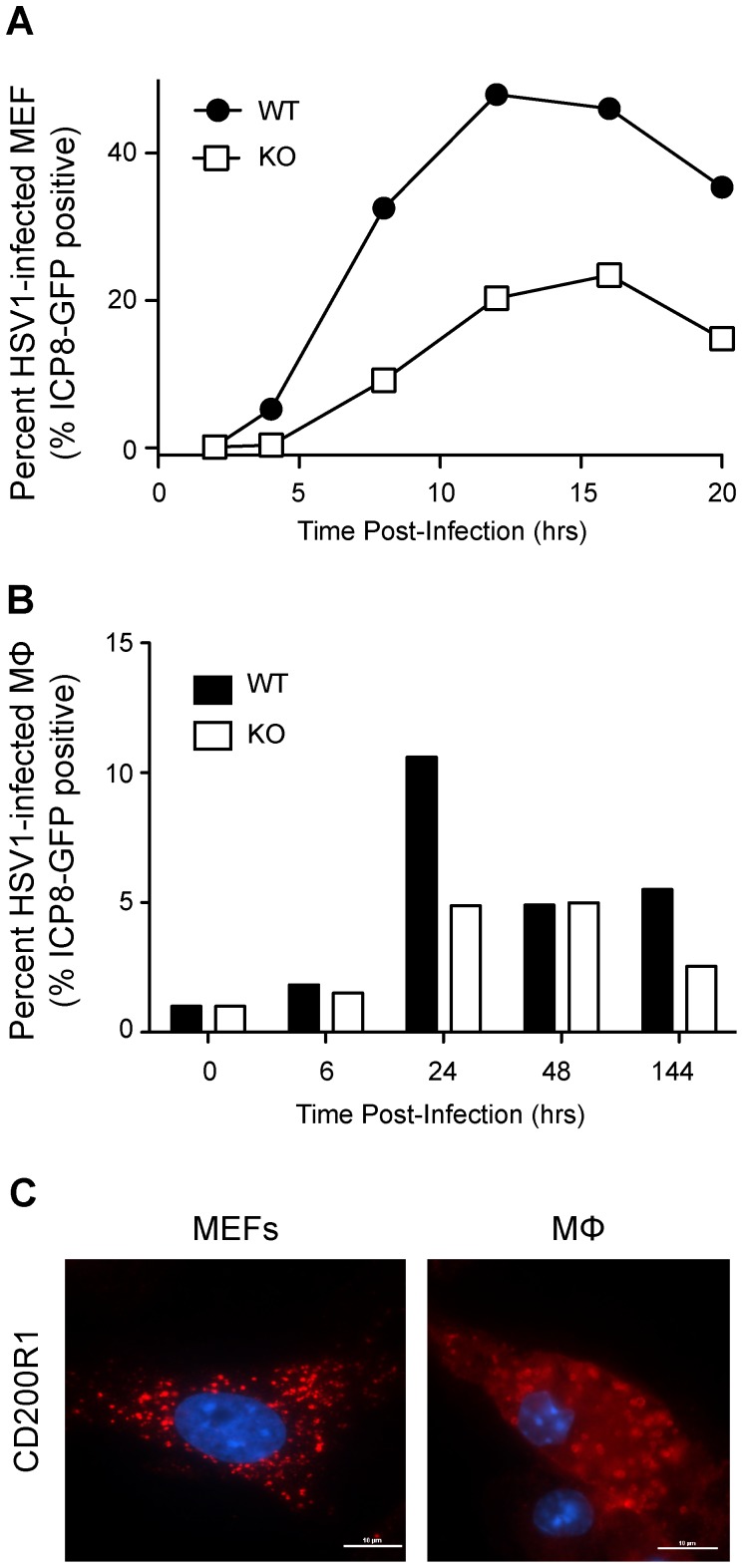
CD200R1 regulates replication of HSV-1 in cells. (A, B) Flow cytometric analysis of GFP expression in CD200R1^+/+^ (WT) and CD200R1^−/−^ (KO) mouse embryonic fibroblasts (MEFs; A) and elicited peritoneal macrophages (B) at various time points following infection with ICP8-GFP virus (MOI 10∶1). Histograms of each sample were generated. A gate was set on the uninfected control for each condition, which excluded 99% of the uninfected cells. This gate was applied to the paired infected samples to calculate the % GFP+ cells. Data representative of 3 experiments. (C) CD200R1 expression on MEFs (left panel) or elicited peritoneal macrophages (MΦ, right panel) was determined using immunofluorescence staining. Scale bar = 10 µm. Data representative of 3–4 experiments (MΦ from n = 6, WT and KO).

To explore the role of CD200R1 in supporting viral replication, we generated mouse embryonic fibroblasts (MEFs) from CD200R1^+/+^ and CD200R1^−/−^ mice and infected them with an HSV-1 strain that expressed a green fluorescent protein (GFP) fusion protein of the delayed early gene ICP8 (ICP8-GFP). We monitored the expression of GFP by flow cytometry for up to 20 h after infection. The expression of ICP8-GFP peaked in CD200R1^+/+^ cells at 12 h after infection, with over 40% of the cells expressing GFP (Figure 6A). In contrast, CD200R1^−/−^ MEFs expressed less than half this amount.

We then tested the ability of CD200R1^−/−^ and CD200R1^+/+^ peritoneal macrophages to be infected with HSV. Macrophages were infected with ICP8-GFP expressing HSV-1 and GFP expression was monitored for up to 144 h. ICP8-GFP expression peaked at 24 h, at which time 10.8% of CD200R1^+/+^ macrophages expressed ICP-GFP. In contrast, only 4.4% of CD200R1^−/−^ macrophages expressed ICP8-GFP (Figure 6B). This was consistent with the results seen in MEFs. Thus HSV-1 infection was attenuated both in CD200R1^−/−^ macrophages and fibroblasts. We confirmed that both elicited peritoneal macrophages and MEFs expressed CD200R1 by immunofluorescence (Figure 6C). Furthermore, neither macrophages nor MEFs express CD200 message or protein (not shown); therefore the impact of CD200R1 on HSV-1 infection was independent of CD200R1:CD200 interaction but was dependent on CD200R1 expression itself. Overall, our findings indicate that CD200R1 plays an important intrinsic role in regulating TLR2 expression and signaling. In a TLR2-dependent viral model like such as HSV-1 infection, lack of CD200R1 expression leads to decreased inflammatory cytokine/chemokine production as well as a failure to upregulate TLR2, thus suggesting a link between TLR2 signaling and decreased viral titers.

## Discussion

The CD200R1:CD200 axis has traditionally been considered to be down-regulatory in inflammatory settings. In models of EAE, *Toxoplasma* encephalitis, experimental autoimmune retinitis, collagen-induced arthritis and influenza-induced pneumonitis, CD200^−/−^ mice show enhanced inflammatory responses [17], [18], [24]. In the case of mouse hepatitis corona virus, the increased survival was attributed to release of inhibition, increased inflammation, and increased viral clearance. Therefore, the dramatic increase in survival of CD200R1^−/−^ mice compared to CD200R1^+/+^ mice in response to intracranial HSV-1 appears to be paradoxical, or potentially explained by the same process. Though a large component of the mortality seen in HSV-1 encephalitis in C57BL/6 WT mice (with an intact type I IFN response) is secondary to the inflammatory response [6], [8], [9], our results emphasize that viral replication, as opposed to inflammation, can also be a key component of the lethal effects of HSV-1 encephalitis in mice. This was supported by the observation that no difference was found between brain chemokine/cytokines levels (with the exception of interferon-β, a marker for viral load) or in leukocyte recruitment into the brains of CD200R1^+/+^ and CD200R1^−/−^ mice. In contrast, our data strongly support a requisite role for CD200R1 in supporting the replication of HSV-1, and implicate decreased viral replication as the reason for increased viability of CD200R1^−/−^ mice. Thus, although brain titers of HSV-1 in CD200R1^+/+^ and CD200R1^−/−^ mice were similar on day 1 post-infection, sustained virus infection was found only in wild type and not in knockout mice. A large proportion of CD200R1^−/−^ mice were essentially free of replicating virus by day 3–4 post-infection in contrast to CD200R1^+/+^ mice which continued to produce infectious HSV-1 virions in their brains over the entire 4 day course of the study.

Our results demonstrate decreased HSV-1 viral titers (in many cases below the limit of detection) and reduced immunohistochemical detection of HSV-1 in the brains of CD200R1^−/−^ mice. Consistent with the reduced levels of brain infection in CD200R1^−/−^ mice we found decreased interferon levels in the brains of CD200R1^−/−^ compared to CD200R1^+/+^ mice. The decreased ability of HSV-1 to infect and/or replicate in tissues outside the brain in CD200R1^−/−^ mice was supported by the blunted HSV-1-specific CD8+ T cell response to intraperitoneal HSV-1 infection. Finally, a role for CD200R1 in supporting viral replication was found by comparing elicited peritoneal macrophages and MEFs from CD200R1^+/+^ and CD200R1^−/−^ mice, in which viral infection was decreased in CD200R1^−/−^ cells.

A unique role for CD200R1 in supporting (licensing) pro-inflammatory signaling by TLR2 was also found. This role was revealed by studies with elicited peritoneal macrophages in which the generation of IL-6 and CCL5 (Rantes) in CD200R1^−/−^ macrophages was blunted by ∼80% in response to TLR2 ligands and HSV-1; this was not observed in response to LPS suggesting a specific defect in TLR2-driven responses. Furthermore, CD200R1^−/−^ macrophages lacked the ability to assemble a functional inflammasome. It is unclear why IL-6 and CCL5 (Rantes) but not MCP-1 levels were reduced in CD200R1^−/−^ macrophages. It may be that there is differential regulation of IL-6 and CCL2 (MCP-1) generation via TLR2 signaling. Although these cytokines are both NF-βB driven, there are distinct transcription factors that drive expression of IL-6 or MCP-1. In previous studies we have noted that HSV-induced IL-6 is strictly dependent on TLR2 expression while some MCP-1 secretion still occurs in TLR2^−/−^ cells, albeit at reduced levels compared to TLR2^+/+^ macrophages [9]. Currently, the mechanism(s) are not known. It is also possible that CD200R1 regulates IL-6 and CCL2 (MCP-1) differently. It is also unclear why the expression of CCL5 (Rantes) but not CCL2 (MCP-1) is decreased in CD200R1^−/−^ mice. It is possible that CCL5 (Rantes) secretion is secondary to decreased IFN, though ultimately this also is a consequence of the control of TLR2 signaling by CD200R1. An additional defect in TLR2 function, the inability to up-regulate the expression of TLR2 in response to HSV-1 infection, may contribute to the inability to perpetuate and amplify HSV-1 infection, though this may be secondary to the role of CD200R1 in regulating viral replication.

The role of CD200R1:CD200 interaction in determining the host response to pathogens is complex, and, when considered in the context of our current studies, is likely to be dependent on the mechanisms that the host uses to kill the specific pathogen. In the case of influenza and meningococcus, CD200^−/−^ mice are prone to the effects of inflammation/sepsis [18], [23], indicating that CD200R1:CD200 interaction is not likely to be involved in the regulation of cellular anti-viral mechanisms. As described above, this is also the case for *Toxoplasma* encephalitis [24]. In regards to Leishmania, redox defense mechanisms used by the host are suppressed by the CD200R1:CD200 axis in *L. amazonensis*, which induces CD200 expression in host cells, so that CD200*^−/−^* mice are protected from the pathogen. However *L. major* grows more slowly in macrophages and does not induce CD200 expression in host tissue [25]. In the case of HSV-1 and other members of the *Herpesviridae* family, the situation is somewhat analogous, but is more complex and involves a clearly distinct mechanism. In HSV-1, CD200R1 supports infection at or before the stage of early gene expression, which in turn regulates the surface expression of TLR2. Whether additional CD200R1-independent or CD200R1-dependent (intrinsic) intracellular mechanisms controlling viral replication are involved are not yet known, but would not be surprising. The decreased infection of HSV-1 at all time points in CD200R1^−/−^ MEFs as well as macrophages indicates that the situation is more complex than simply the suppression of a virus-toxic metabolite such as NO, which can be driven or amplified by TLR2. The effect of CD200R1 on HSV-1 infection in MEFs and macrophages occurred in the absence CD200 expression suggesting that CD200R1 intrinsically regulates virus replication. Because HSV-1 is a neurotropic virus and neurons express CD200 but not CD200R1 on their surface [50], [51], how the macrophage-specific mechanisms elucidated by our work function to exert their profound impact on HSV-1 replication and host survival *in vivo* are not straightforward. Even though macrophages and dendritic cells are relatively resistant to HSV-1 infection [52], our data indicate that they have the potential to be infected, and therefore could be important in mediating intraneuronal transmission or to serve as a reservoir of virus. As CD200R1^−/−^ macrophages would be deficient in these processes, the lack of CD200R1 could potentially slow the spread of virus within the brain. Alternatively or concomitantly, CD200R1^+/+^ macrophages may generate a factor that is required to sustain viral replication in neurons, or CD200R1^−/−^ macrophages may contribute to increased viral killing. Furthermore, how the impact of CD200R1 on TLR2 signaling affects viral replication *in vivo* is not fully elucidated. HSV-1 is known to target components of the TLR2 signaling pathway via expression of the viral protein ICP0, suggesting that TLR2 biology and HSV-1 biology are linked [53].

The selective requirement for the induction of IL-6 and CCL5 (Rantes) expression by TLR2 ligands and HSV-1 is consistent with the emerging concept that inhibitory receptors can be multifunctional and be required for pro-inflammatory signaling. CD150 contains a TxYxxV/I motif in its cytoplasmic tail that can bind SH2-containing molecules, ranging from tyrosine and inositol phosphatases and Src family kinases to adaptor molecules. The function of CD150 can vary depending on the molecule with which it interacts [27]. CD200R1 has neither a conventional ITIM nor an ITSM domain, and the range of potential interactions with its cytoplasmic tail is not known. Another example of a dual role for inhibitory receptors is the fact that for their activation receptors to become activated, NK cell inhibitory receptors must first interact with their cognate self-MHC ligands [54]. In fact, the role of CD200R1 in supporting the synthesis of IL-6 and CCL5 (Rantes) may not be direct. The blunted generation of IL-6 and CCL5 (Rantes) was determined after 24 h, so signaling by CD200R1 could impact the synthesis of other cytokines, which in turn regulate the generation of IL-6 and CCL5 (Rantes). However, the role of CD200R1 in regulating the surface expression of TLR2 expression suggests a direct link between these pathways.

## Supporting Information

Figure S1 **Gene Targeting of CD200R1.** (A) Development of the targeting construct. (B) Southern blot of tail vein DNA from CD200R1^−/−^ (−/−), CD200R1^+/+^ (+/+), and CD200R1^+/−^ (+/−) mice. The 7.8 kbp band indicates the WT allele, whereas the 2.8 kbp band is diagnostic of a gene-targeted allele. (C) PCR analysis of tail vein DNA from CD200R1^−/−^ (−/−), CD200R1^+/+^ (+/+), and CD200R1^+/−^(+/−) mice. The gene-targeted allele is represented by a 1.5 kbp band and the WT allele is represented by a 1.4 kbp band. (D) Flow cytometric analysis of elicited peritoneal macrophages from CD200R1^+/+^ (WT) and CD200R1^−/−^ (KO) macrophages probed for the expression of CD200R1.
(PDF)
