# Supplementary figures and images for: CD200R1 Supports HSV-1 Viral Replication and Licenses Pro-Inflammatory Signaling Functions of TLR2
Source: PLoS One. 2012 Oct 17;7(10):e47740. doi: 10.1371/journal.pone.0047740 (PMC3474780; doi:10.1371/journal.pone.0047740)

Supporting Figure S1

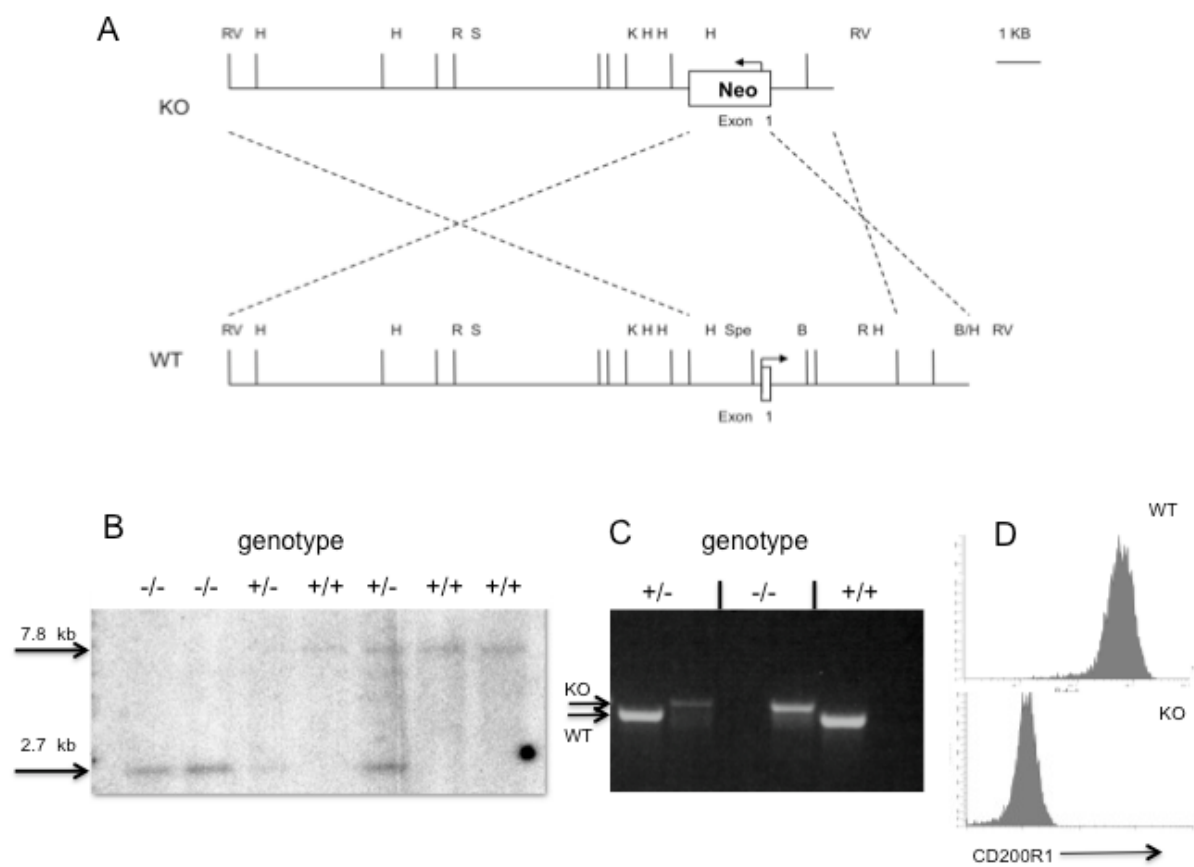

Supplement: Figure S1 — Gene Targeting of CD200R1. (A) Development of the targeting construct. (B) Southern blot of tail vein DNA from CD200R1−/− (−/−), CD200R1+/+ (+/+), and CD200R1+/− (+/−) mice. The 7.8 kbp band indicates the WT allele, whereas the 2.8 kbp band is diagnostic of a gene-targeted allele. (C) PCR analysis of tail vein DNA from CD200R1−/− (−/−), CD200R1+/+ (+/+), and CD200R1+/−(+/−) mice. The gene-targeted allele is represented by a 1.5 kbp band and the WT allele is represented by a 1.4 kbp band. (D) Flow cytometric analysis of elicited peritoneal macrophages from CD200R1+/+ (WT) and CD200R1−/− (KO) macrophages probed for the expression of CD200R1. (PDF) [file pone.0047740.s001.pdf]
